# Performance of Modified Early Warning Score (MEWS) for Predicting In-Hospital Mortality in Traumatic Brain Injury Patients

**DOI:** 10.3390/jcm10091915

**Published:** 2021-04-28

**Authors:** Dong-Ki Kim, Dong-Hun Lee, Byung-Kook Lee, Yong-Soo Cho, Seok-Jin Ryu, Yong-Hun Jung, Ji-Ho Lee, Jun-Ho Han

**Affiliations:** Department of Emergency Medicine, Chonnam National University Medical School, 160 Baekseo-ro, Dong-gu, Gwangju 61469, Korea; lifelorddg@naver.com (D.-K.K.); bbukkuk@hanmail.net (B.-K.L.); semi-moon@hanmail.net (Y.-S.C.); samahalak@naver.com (S.-J.R.); xnxn77@hanmail.net (Y.-H.J.); rake21c@naver.com (J.-H.L.); ckris12345@naver.com (J.-H.H.)

**Keywords:** traumatic brain injury, scoring system, modified early warning score, mortality

## Abstract

The present study aimed to analyze and compare the prognostic performances of the Revised Trauma Score (RTS), Injury Severity Score (ISS), Shock Index (SI), and Modified Early Warning Score (MEWS) for in-hospital mortality in patients with traumatic brain injury (TBI). This retrospective observational study included severe trauma patients with TBI who visited the emergency department between January 2018 and December 2020. TBI was considered when the Abbreviated Injury Scale was 3 or higher. The primary outcome was in-hospital mortality. In total, 1108 patients were included, and the in-hospital mortality was 183 patients (16.3% of the cohort). Receiver operating characteristic curve analyses were performed for the ISS, RTS, SI, and MEWS with respect to the prediction of in-hospital mortality. The area under the curves (AUCs) of the ISS, RTS, SI, and MEWS were 0.638 (95% confidence interval (CI), 0.603–0.672), 0.742 (95% CI, 0.709–0.772), 0.524 (95% CI, 0.489–0.560), and 0.799 (95% CI, 0.769–0.827), respectively. The AUC of MEWS was significantly different from the AUCs of ISS, RTS, and SI. In multivariate analysis, age (odds ratio (OR), 1.012; 95% CI, 1.000–1.023), the ISS (OR, 1.040; 95% CI, 1.013–1.069), the Glasgow Coma Scale (GCS) score (OR, 0.793; 95% CI, 0.761–0.826), and body temperature (BT) (OR, 0.465; 95% CI, 0.329–0.655) were independently associated with in-hospital mortality after adjustment for confounders. In the present study, the MEWS showed fair performance for predicting in-hospital mortality in patients with TBI. The GCS score and BT seemed to have a significant role in the discrimination ability of the MEWS. The MEWS may be a useful tool for predicting in-hospital mortality in patients with TBI.

## 1. Introduction

Trauma is the leading cause of death in people aged below 46 years [1]. Although the mortality of trauma patients has declined over the last decades, the cause of trauma-related death has gradually shifted from multiple organ dysfunction syndrome to central nervous injury [2]. Therefore, it is important to identify risk factors early and provide intensive care for patients with traumatic brain injury (TBI).

Several triage tools for TBI have been developed, and studies have reported the efficacies of these tools for predicting prognosis [3,4,5,6,7,8]. Among these, the Injury Severity Score (ISS) and Revised Trauma Score (RTS) are the most commonly used tools in severe trauma patients, including those with TBI [3,4]. However, the relationship between these tools and the prognosis of patients with TBI is not well understood, and some studies have even questioned these relationships [9,10,11]. The Shock Index (SI), the ratio of heart rate to systolic blood pressure (SBP), was related to hypovolemic shock in patients with severe trauma, including TBI [5,6], and may be related to the mortality of patients with TBI [7]. In addition, previous studies have reported that early warning scores, such as the Modified Early Warning Score (MEWS), are related to adverse events, including hypotension and the need for advanced airway management, need for intensive care, and early mortality in patients with TBI [8]. However, few studies have shown the association between various triage tools and outcomes in patients with TBI.

Therefore, this study aimed to analyze and compare the prognostic performances of the RTS, ISS, SI, and MEWS for in-hospital mortality in patients with TBI. We also investigated the risk factors associated with in-hospital mortality in patients with TBI.

## 2. Materials and Methods

### 2.1. Study Design and Population

We performed a retrospective observational study involving patients with TBI at Chonnam National University Hospital, Gwangju, South Korea, who were admitted between January 2018 and December 2020. Severe trauma was defined as an ISS greater than 15 [12]. TBI was considered when the head Abbreviated Injury Scale (AIS) score was 3 or higher [13]. Isolated TBI was defined as a head AIS score of ≥3 and any other AIS score of <3 [14]. Combined TBI was defined as a head AIS score of ≥3 and at least one other AIS score of ≥3 [14]. The following exclusion criteria were applied: age below 18 years; cardiac arrest following trauma before arrival at the emergency department (ED); specific trauma mechanisms, such as drowning, burns, or hanging; and missing data. This study was approved by the institutional review board of Chonnam National University Hospital (CNUH-2021-064).

Vital sign and Glasgow Coma Scale (GCS) scores were measured by triage nurses who have received in-hospital education and training in the triage room at ED visits. All the triage nurses have been working in the ED for at least 2 years before performing triage. The AIS and ISS scores were calculated by physicians who have received training in Korean Trauma Assessment and Treatment (KTAT).

### 2.2. Data Collection

Data on the following variables were obtained for each patient: age, sex, mechanism of trauma, SBP (mmHg) on admission, respiratory rate on admission, pulse rate on admission, body temperature (BT, °C) on admission, initial Glasgow Coma Scale (GCS) score, amount of transfused packed red blood cells (PRC), fresh frozen plasma (FFP), and platelet concentrates (PC) within 24 h after arrival at the ED, and in-hospital mortality.

The RTS was calculated based on vital signs and the GCS score (Table 1) [15]. The SI was calculated as the heart rate divided by SBP [5]. The AIS score and ISS were calculated on ED arrival. The MEWS was calculated based on vital signs and AVPU (Alert, Voice, Pain, Unresponsive) scale data on ED arrival (Table 2) [16]. The primary outcome was in-hospital mortality.

### 2.3. Statistical Analysis

Continuous variables did not satisfy the normality test and are presented as median values with interquartile ranges (IQR). Categorical variables are presented as frequencies and percentages. Differences between survivors and non-survivors were tested using the Mann-Whitney *U*-test for continuous variables. Fisher’s exact test or the chi-square test was used for the comparison of categorical variables, as appropriate. Receiver operating characteristic (ROC) curve analysis was performed to examine the prognostic performances of the ISS, RTS, SI, and MEWS for in-hospital mortality. The comparison of dependent ROC curves was performed using the DeLong method [17].

We conducted multivariate analysis using logistic regression of relevant covariates for in-hospital mortality. Variables with p values of <0.20 in univariate comparisons were included in the multivariate regression model. We used a backward stepwise approach, sequentially eliminating variables with a threshold p value of >0.10 to build the final adjusted regression model. We included one of the prognostic tools (MEWS, RTS, ISS, and SI) into the final model and performed the analysis separately in each group (all TBI, isolated TBI, and combined TBI groups). The results of logistic regression analysis are presented as odds ratios (ORs) and 95% confidence intervals (CIs). All analyses were performed using PASW/SPSS™ software, version 18 (IBM Inc., Chicago, IL, USA) and MedCalc version 19.0 (MedCalc Software, bvba, Ostend, Belgium). A two-sided significance level of 0.05 was used to indicate statistical significance.

## 3. Results

### 3.1. Patient Selection and Characteristics

**Figure 1:**
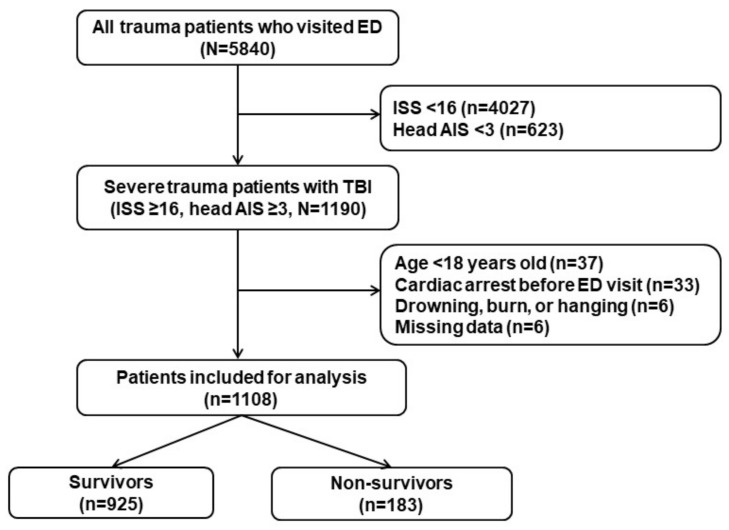
Schematic diagram showing the number of patients with TBI in the present study. TBI, traumatic brain injury; ISS, Injury Severity Score; AIS, Abbreviated Injury Scale.

In total, 1190 severe trauma patients were identified during the study period who met the inclusion criteria. Based on the exclusion criteria, 1108 patients were finally included in this study (Figure 1). There were 822 (74.2%) male patients, and the median age was 64.1 years (53.0–75.0 years). The in-hospital mortality rate was 16.5% (*n* = 183).

### 3.2. Comparison of Baseline and Clinical Characteristics between Survivors and Non-Survivors

Table 3 shows the comparison of baseline and clinical characteristics between survivors and non-survivors. Survivors had higher RTS, GCS score, and BT values and lower ISS, pulse rate, and SI values. SBP was not significantly different between survivors and non-survivors. The proportion of patients with hypothermia among non-survivors was higher than that among survivors. The MEWS (2 (1–3) vs. 5 (4–6); *p* < 0.001) was significantly lower in survivors than in non-survivors.

In the isolated TBI group, survivors had higher RTS, GCS score, and BT values and lower ISS and PR values than non-survivors. The MEWS (2 (1–3) vs. 4 (3–6); *p* < 0.001) was significantly lower in survivors than in non-survivors (Table 4).

In the combined TBI group, survivors had higher RTS, GCS score, SBP, and BT values and lower ISS and SI values than non-survivors. The MEWS (2 (1–4) vs. 6 (5–7); *p* < 0.001) was significantly lower in survivors than in non-survivors (Table 4).

### 3.3. Prognostic Performance of the ISS, RTS, SI, and MEWS for in-Hospital Mortality

**Figure 2:**
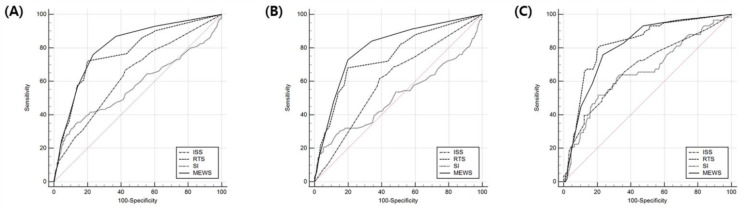
Receiver operating characteristic curve analyses of the ISS, RTS, SI, and MEWS for predicting in-hospital mortality. (**A**) Total TBI group: the AUCs of the ISS, RTS, SI, and MEWS were 0.638 (95% CI, 0.603–0.672), 0.742 (95% CI, 0.709–0.772), 0.524 (95% CI, 0.489–0.560), and 0.799 (95% CI, 0.769–0.827), respectively. (**B**) Isolated TBI group: the AUCs of the ISS, RTS, SI, and MEWS were 0.608 (95% CI, 0.574–0.641), 0.750 (95% CI, 0.719–0.778), 0.510 (95% CI, 0.476–0.544), and 0.803 (95% CI, 0.774–0.829), respectively. (**C**) Combined TBI group: the AUCs of the ISS, RTS, SI, and MEWS were 0.679 (95% CI, 0.619–0.735), 0.824 (95% CI, 0.773–0.868), 0.657 (95% CI, 0.597–0.715), and 0.809 (95% CI, 0.757–0.855), respectively. ISS, Injury Severity Score; RTS, Revised Trauma Score; SI, Shock Index; MEWS, Modified Early Warning Score; TBI, traumatic brain injury; AUC, area under curve; CI, confidence interval

The areas under the curve (AUCs) of the ISS, RTS, SI, and MEWS for predicting in-hospital mortality were 0.638 (95% CI, 0.603–0.672), 0.742 (95% CI, 0.709–0.772), 0.524 (95% CI, 0.489–0.560), and 0.799 (95% CI, 0.769–0.827), respectively (Figure 2A). 

The AUC of the MEWS was significantly different from the AUCs of the ISS, RTS, and SI (Table 5).

In the isolated TBI group, the AUCs of the ISS, RTS, SI, and MEWS for predicting in-hospital mortality were 0.608 (95% CI, 0.574–0.641), 0.750 (95% CI, 0.719–0.778), 0.510 (95% CI, 0.476–0.544), and 0.803 (95% CI, 0.774–0.829), respectively (Figure 2B). The AUC of the MEWS in the isolated TBI group was significantly different from the AUCs of the ISS, RTS, and SI (Table 5).

In the combined TBI group, the AUCs of the ISS, RTS, SI, and MEWS for predicting in-hospital mortality were 0.679 (95% CI, 0.619–0.735), 0.824 (95% CI, 0.773–0.868), 0.657 (95% CI, 0.597–0.715), and 0.809 (95% CI, 0.757–0.855), respectively (Figure 2C). The AUC of the MEWS in the combined TBI group was significantly different from the AUCs of the ISS and SI but not from the AUC of the RTS (Table 5).

### 3.4. Multivariate Logistic Regression Analysis for in-Hospital Mortality

Table 6 shows the results of the multivariate analysis performed for in-hospital mortality. In all TBI group, age (OR, 1.013; 95% CI, 1.001–1.025), low GCS score (OR, 0.86; 95% CI, 0.54–0.820), low BT (OR, 0.537; 95% CI, 0.382–0.753), FFP (OR, 1.216; 95% CI, 1.129–1.310), and PC (OR, 1.018; 95% CI, 1.000–1.037) were independently associated with in-hospital mortality. In the isolated TBI group, low GCS score (OR, 0.792; 95% CI, 0.754–0.831), low BT (OR, 0.574; 95% CI, 0.398–0.830), FFP (OR, 1.226; 95% CI, 1.100–1.367), and PC (OR, 1.026; 95% CI, 1.002–1.049) were independently associated with in-hospital mortality (Table 6); while in the combined TBI group, age (OR, 1.033; 95% CI, 1.007–1.060), low GCS score (OR, 0.759; 95% CI, 0.698–0.824), low BT (OR, 0.424; 95% CI, 0.186–0.965), and PRC (OR, 1.153; 95% CI, 1.061–1.254) were independently associated with in-hospital mortality (Table 6).

Among the prognostic tools assessed, MEWS and RTS were associated with in-hospital mortality in all TBI, isolated TBI, and combined TBI groups, after adjusting for confounders (Table 7). ISS and SI were not associated with in-hospital mortality in all TBI, isolated TBI, and combined TBI groups.

## 4. Discussion

In the present study, the MEWS showed fair performance for predicting in-hospital mortality in patients with TBI. The GCS score and BT were associated with in-hospital mortality in all groups, including the total TBI, isolated TBI, and combined TBI groups.

The SI (the ratio of heart rate to SBP) showed poor performance for predicting in-hospital mortality in the present study. It was assumed that in all groups, SBP and heart rate had no relationship with the mortality of patients with TBI. McMahon et al. showed that the SI responded later to hemorrhage in the TBI group compared to the non-TBI group, and responded later in non-survivors compared to survivors [18]. Moreover, factors such as medication for hypertension and beta blockers can modulate SI at the compensation stage of the shock. The ISS was not associated with in-hospital mortality in all TBI, isolated TBI, and combined TBI groups. An important disadvantage of the ISS is that only one injury is considered in each body part. Since TBI patients with head AIS score of ≥ 3 were included in the present study, other injuries could have been overlooked. In contrast, previous studies have reported the association of ISS with mortality in TBI patients [19,20]. Thus, further research may be needed to clarify the relationship between ISS and prognosis of TBI. In this study, the RTS and MEWS were related to the mortality of patients with TBI. A previous study revealed that the RTS was related to the mortality of patients with TBI [20], and the MEWS was also likely to be related to the outcomes of patients with TBI in other studies [8,21]. As both the RTS and MEWS include the GCS score, which was associated with the prognosis of TBI, they were expected to show good performance for predicting mortality. However, the MEWS showed better performance than the RTS in the total TBI and isolated TBI groups in the present study. In our study, BT was associated with mortality in all groups. As the MEWS includes BT, which is not included in the RTS, the MEWS would be more accurate in predicting mortality than the RTS. In addition, since RTS includes GCS, there may be difficulties in measuring RTS when compared to measurements of MEWS, including AVPU. In particular, it is challenging to measure GCS-motor or GCS-verbal of intubated patients.

Several studies have demonstrated that the GCS score was related to the mortality of patients with TBI [3,22]. In a study by Han et al., a GCS score of ≤5 was associated with mortality in most groups, and the GCS score of non-survivors was 4 (3–9) in this study [22]. In another study on patients with TBI, the OR of the GCS score for mortality was 0.765, similar to that obtained in the present study [3], in which the GCS score of non-survivors corresponded to the unresponsiveness parameter in the AVPU scale [23]. Thus, it corresponded to 3 points in the MEWS and was believed to have played an important role in the performance of the MEWS [16].

Previous studies have revealed that a low BT was associated with mortality in patients with TBI [24,25,26]. In patients with severe trauma, including patients with TBI, bleeding caused hypovolemia, which can lead to lower BT; this accelerates coagulation disorders and eventually affects prognosis [27]. In contrast, low BT at the time of ED visit was related to mortality, even though the major injury was limited to a head injury, such as isolated TBI, in the present study. In other studies on isolated TBI, low BT at admission was associated with mortality [28,29]. This can be explained by the fact that a low BT at admission in patients with TBI reflects severe head injury. De Tanti et al. speculated that hypothalamic dysfunction due to brain injury may contribute to mortality in patients with severe TBI [30].

In the present study, the SBP of patients with isolated TBI was not associated with in-hospital mortality. A previous study also showed that SBP may be insufficient to predict the mortality of patients with TBI [31]. This could be attributed to the effect of cerebral autoregulation in patients with TBI with elevated intracranial pressure (ICP). Cerebral autoregulation is a homeostatic process that regulates and maintains cerebral blood flow across a range of blood pressures [32]. Thus, the elevation of ICP increases arterial blood pressure to maintain the perfusion pressure to the brain [33]. In contrast, SBP was associated with in-hospital mortality in the combined TBI group in the present study. The reason for this may be the difference in SBP between the combined TBI (110 (90–130) mmHg) and isolated TBI (130 (110–150) mmHg) groups. The combined TBI included bleeding from other body regions, such as the head, as well as head injury; thus, SBP would be lower in the combined TBI group than in the isolated TBI group. In a study of patients with TBI, including those with combined TBI, mortality increased when the SBP dropped from 110 to 100 mmHg [34].

This study had several limitations. First, it was a retrospective study that was performed at a single center. Therefore, its findings are not immediately generalizable to the overall population. Further multi-center studies with larger sample sizes and prospective designs are needed to substantiate our findings. Second, we did not analyze the effects of essential procedures (such as interventions, operations, and transfusions) on in-hospital mortality. Further research is needed to address these effects. Third, the measurements for vital signs and GCS scores may be inconsistent and vary from person-to-person. Although triage nurses have been constantly educated and trained, the results may be affected by individual medical experience. Fourth, we did not specifically record the site of temperature measurement as BT can vary depending on the region of the body. Thus, this may be considered as a confounder to our data analyses. Fifth, we did not consider the natural circadian rhythm of body temperature, although these effects would be limited during acute illnesses, such as TBI [35]. Sixth, the patient’s clinical condition, such as the effects of comorbidities and drugs, was not investigated. Since such conditions can affect the patient’s prognosis, these factors should be included in future research. Finally, we did not investigate the cause of death in patients with TBI. The most common causes of trauma-related death are central nervous injury and blood loss, and we did not compare and analyze the relationship between these causes and the various prediction tools, including the MEWS.

## 5. Conclusions

In the present study, the MEWS showed fair performance for predicting in-hospital mortality in patients with TBI. The GCS score and BT seemed to have a significant role in the discrimination ability of the MEWS. Therefore, the MEWS may be a useful tool for predicting in-hospital mortality in patients with TBI.

## Figures and Tables

**Table 1:** Revised Trauma Score.

The Revised Trauma Score (RTS)			
Glasgow Coma Scale (GCS)	Systolic Blood Pressure (SBP)	Respiratory Rate (RR)	Coded Value
13–15	>89	10–29	4
9–12	76–89	>29	3
6–8	50–75	6–9	2
4–5	1–49	1–5	1
3	0	0	0

RTS = 0.9368 (GCSc) + 0.7326 (SBPc) + 0.2908 (RRc).

**Table 2:** Modified Early Warning Score.

Modified Early Warning Score (MEWS)				
Score	0	1	2	3
Respiratory rate (min^−1^)	9–14	15–20	21–29	≥ 30
			≤ 8	
Hear rate (min^−1^)	51–100	101–110	111–129	≥ 130
		41–50	≤ 40	
Systolic BP (mmHg)	101–199		≥ 200	
		81–100	71–80	≤ 70
Temperature (°C)	35.1–38.4		≥ 38.5	
			≤ 35	
Neurological	Alert	Responding to Voice	Responding to Pain	Unresponsive

The total score is the sum of each component.

**Table 3:** Comparison of baseline characteristics of TBI patients according to in-hospital mortality.

Variables	TBI Patients (*N* = 1108)	Survivors (*N* = 925)	Non-Survivors (*N* = 183)	*p* Value
Age, years, IQR	64.1 (53.0–75.0)	64.0 (53.0–75.0)	67.0 (53.0–76.1)	0.199
Male, n (%)	822 (74.2)	683 (73.8)	139 (76.0)	0.550
Mechanism of trauma				0.416
Blunt, n (%)	1,103 (99.5)	922 (99.7)	181 (98.9)	
Penetrating, n (%)	5 (0.5)	3 (0.3)	2 (1.1)	
Revised Trauma Score, IQR	5.97 (5.03–7.84)	5.97 (5.64–7.84)	4.09 (2.83–5.64)	<0.001
Injury Severity Score, IQR	22 (16–25)	21 (16–25)	25 (20–29)	<0.001
Glasgow Coma Scale, IQR	14 (7–15)	15 (10–15)	4 (3–9)	<0.001
Systolic BP, mmHg, IQR	130 (110–140)	130 (110–140)	120 (90–160)	0.050
Respiratory rate, /min, IQR	20 (20–20)	20 (20–20)	20 (20–22)	0.022
Pulse rate, /min, IQR	84 (74–96)	84 (74–94)	90 (72–104)	0.006
BT, °C, IQR	36.4 (36.1–36.7)	36.4 (36.2–36.8)	36.2 (36.0–36.5)	<0.001
BT ≤35 °C, n (%)	44 (4.0)	17 (1.8)	27 (14.8)	<0.001
PRC, unit	0 (0–2)	0 (0–1)	6 (5–12)	<0.001
FFP, unit	0 (0–2)	0 (0–0)	4 (2–8)	<0.001
PC, unit	0 (0–0)	0 (0–0)	6 (0–10)	<0.001
Shock Index	0.65 (0.54–0.82)	0.65 (0.54–0.80)	0.69 (0.54–1.13)	0.002
MEWS	2 (1–4)	2 (1–3)	5 (4–6)	<0.001

TBI, traumatic brain injury; IQR, interquartile range; BP, blood pressure; BT, body temperature; PRC, packed red blood cell; FFP, fresh frozen plasma; PC, platelet concentrates; MEWS, Modified Early Warning Score.

**Table 4:** Comparison of baseline characteristics according to in-hospital mortality in isolated TBI and combined TBI groups.

Variables	Isolated TBI (*N* = 845)		Combined TBI (*N* = 263)			
	Survivors (*N* = 720)	Non-Survivors (*N* = 125)	*p* Value	Survivors (*N* = 205)	Non-Survivors (*N* = 58)	*p* Value
Age, years, IQR	65 (54–75)	67 (53–78)	0.366	60 (50–71)	65 (53–74)	0.104
Male, n (%)	533 (74.0)	93 (74.4)	1.000	150 (73.2)	46 (79.3)	0.437
Mechanism of trauma			0.927			0.920
Blunt, n (%)	718 (99.7)	124 (99.2)		204 (99.5)	57 (98.3)	
Penetrating, n (%)	2 (0.3)	1 (0.8)		1 (0.5)	1 (1.7)	
ISS, IQR	17 (16–25)	25 (16–25)	<0.001	25 (22–29)	31 (25–38)	<0.001
RTS, IQR	5.97 (5.64–7.84)	4.09 (2.83–5.97)	<0.001	6.38 (5.64–7.84)	4.09 (2.83–5.23)	<0.001
GCS, IQR	14 (9–15)	4 (3–10)	<0.001	15 (10–15)	4 (3–8)	<0.001
SBP, mmHg, IQR	130 (110–150)	140 (100–160)	0.224	110 (100–130)	90 (70–110)	<0.001
RR, /min, IQR	20 (20–20)	20 (20–22)	0.199	20 (20–22)	20 (20–24)	0.086
PR, /min, IQR	82 (72–92)	87 (71–103)	0.046	90 (79–104)	96 (76–110)	0.237
BT, °C, IQR	36.4 (36.2–36.8)	36.2 (36.0–36.5)	<0.001	36.4 (36.1–36.8)	36.2 (36.0–36.4)	<0.001
PRC, unit	0 (0-0)	1 (0–4)	<0.001	2 (0–4)	4 (2–10)	<0.001
FFP, unit	0 (0-0)	0 (0–2)	<0.001	0 (0–2)	3 (0–8)	<0.001
PC, unit	0 (0-0)	0 (0–0)	<0.001	0 (0–0)	0 (0–0)	<0.001
SI, IQR	0.62 (0.53–0.74)	0.63 (0.49–0.87)	0.726	0.81 (0.64–1.00)	1.09 (0.73–1.38)	<0.001
MEWS, IQR	2 (1–3)	4 (3–6)	<0.001	2 (1–4)	6 (5–7)	<0.001

TBI, traumatic brain injury; IQR, interquartile range; ISS, Injury Severity Score; RTS, Revised Trauma Score; GCS, Glasgow Coma Scale; SBP, systolic blood pressure; RR, respiratory rate; PR, pulse rate; BT, body temperature; PRC, packed red blood cell; FFP, fresh frozen plasma; PC, platelet concentrates; SI, Shock Index; MEWS, Modified Early Warning Score.

**Table 5:** Pairwise comparison test of the ROC curves including MEWS, RTS, ISS, and SI for in-hospital mortality in TBI patients.

	Difference between Areas	SE	95% CI	*p* Value
All TBI group				
MEWS vs. RTS	0.0575	0.0218	0.0147 to 0.100	0.0085
MEWS vs. ISS	0.161	0.0297	0.103 to 0.219	<0.0001
MEWS vs. SI	0.275	0.0311	0.214 to 0.336	<0.0001
RTS vs. ISS	0.104	0.0341	0.0368 to 0.170	0.0024
RTS vs. SI	0.217	0.0386	0.142 to 0.293	<0.0001
ISS vs. SI	0.114	0.0403	0.0347 to 0.193	0.0048
Isolated TBI group				
MEWS vs. RTS	0.0532	0.0217	0.0106 to 0.0958	0.0144
MEWS vs. ISS	0.195	0.0301	0.136 to 0.254	<0.0001
MEWS vs. SI	0.293	0.0324	0.229 to 0.356	<0.0001
RTS vs. ISS	0.142	0.0332	0.0770 to 0.207	<0.0001
RTS vs. SI	0.240	0.0390	0.163 to 0.316	<0.0001
ISS vs. SI	0.0976	0.0444	0.0107 to 0.185	0.0278
Combined TBI group				
MEWS vs. RTS	0.0147	0.0277	−0.0397 to 0.0691	0.5957
MEWS vs. ISS	0.130	0.0433	0.0453 to 0.215	0.0026
MEWS vs. SI	0.152	0.0350	0.0834 to 0.221	<0.0001
RTS vs. ISS	0.145	0.0445	0.0575 to 0.232	0.0011
RTS vs. SI	0.167	0.0479	0.0728 to 0.261	0.0005
ISS vs. SI	0.0220	0.0591	−0.0939 to 0.138	0.7104

MEWS, Modified Early Warning Score; RTS, Revised Trauma Score; ISS, Injury Severity Score; SI, Shock Index; ROC, receiver operator characteristic; SE, standard error; CI, confidence interval.

**Table 6:** Multivariate logistic regression analysis for predicting in-hospital mortality in TBI patients.

	All TBI Group		Isolated TBI Group		Combined TBI Group	
	Adjusted OR (95% CI)	*p* Value	Adjusted OR (95% CI)	*p* Value	Adjusted OR (95% CI)	*p* Value
Age, years	1.013(1.001-1.025)	0.036			1.033 (1.007–1.060)	0.014
GCS score	0.786 (0.754–0.820)	<0.001	0.792 (0.754–0.831)	<0.001	0.759 (0.698–0.824)	<0.001
SBP, mmHg	1.002 (0.997–1.008)	0.428			1.003 (0.992–1.013)	0.616
RR, /min	1.038 (0.966–1.115)	0.315	1.020 (0.931–1.119)	0.670	1.086 (0.965–1.221)	0.173
PR, /min	1.006 (0.997–1.015)	0.203	1.006 (0.995–1.017)	0.324		
BT, °C	0.537 (0.382–0.753)	<0.001	0.574 (0.398–0.830)	0.003	0.424 (0.186–0.965)	0.041
PRC, unit	0.988 (0.897–1.087)	0.802	0.922 (0.814–1.043)	0.196	1.153 (1.061–1.254)	0.001
FFP, unit	1.216 (1.129–1.310)	<0.001	1.226 (1.100–1.367)	<0.001	1.047 (0.853–1.285)	0.661
PC, unit	1.018 (1.000–1.037)	0.048	1.026 (1.002–1.049)	0.030	1.002 (0.969–1.036)	0.914

TBI, traumatic brain injury; OR, odds ratio; CI, confidence interval; GCS, Glasgow Coma Scale; SBP, systolic blood pressure; RR, respiratory rate; PR, pulse rate; BT, body temperature; PRC packed red blood cell; FFP fresh frozen plasma; PC, platelet concentrates.

**Table 7:** Multivariate logistic regression analysis of MEWS, RTS, ISS, and SI for predicting in-hospital mortality in TBI ^1^ patients.

	All TBI Group		Isolated TBI Group		Combined TBI Group	
	Adjusted OR (95% CI)	*p* Value	Adjusted OR (95% CI)	*p* Value	Adjusted OR (95% CI)	*p* Value
MEWS	1.605 (1.470–1.753) ^1^	<0.001	1.695 (1.519–1.891) ^4^	<0.001	1.515 (1.302–1.762) ^7^	<0.001
RTS	0.594 (0.534–0.659) ^2^	<0.001	0.614 (0.544–0.693) ^5^	<0.001	0.513 (0.408–0.644) ^8^	<0.001
ISS	1.014 (0.984–1.045) ^3^	0.357	1.015 (0.967–1.067) ^6^	0.543	1.013 (0.964–1.065) ^9^	0.605
SI	1.385 (0.840–2.282) ^3^	0.202	1.479 (0.769–2.843) ^6^	0.241	1.143 (0.469–2.787) ^9^	0.769

Each prognostic tool was individually entered into the final model and analyzed separately. Each prognostic tool was not adjusted for other tools. TBI, traumatic brain injury; OR, odds ratio; CI, confidence interval; MEWS, Modified Early Warning Score; RTS, Revised Trauma Score; ISS, Injury Severity Score; SI, Shock Index; PRC packed red blood cell; FFP fresh frozen plasma; PC, platelet concentrates; GCS, Glasgow Coma Scale; BT, body temperature. ^1^ Adjusted for age, FFP, and PC. ^2^ Adjusted for age, BT, FFP, and PC. ^3^ Adjusted for age, GCS, BT, FFP, and PC. ^4^ Adjusted for FFP, and PC. ^5^ Adjusted for BT, FFP, and PC. ^6^ Adjusted for GCS, BT, FFP, and PC. ^7^ Adjusted for age and PRC. ^8^ Adjusted for age, BT, and PRC. ^9^ Adjusted for age, GCS, BT, and PRC.

## Data Availability

The data presented in this study are available on request from the corresponding author. The data are not publicly available due to personal protection.
